# The Transition to Independence: A Longitudinal Qualitative Study on Drivers of Burnout in Japanese Early‐Career Physicians

**DOI:** 10.1002/jgf2.70159

**Published:** 2026-08-02

**Authors:** Mitsuru Watanabe, Kosuke Nakano, Yui Amari, Yukina Yokoyama, Mizuki Kato, Yoichiro Haji

**Affiliations:** ^1^ Department of Rheumatology Daido Hospital Nagoya City Aichi Japan

## Abstract

**Background/Objective:**

The transition from postgraduate residency to independent practice is a critical period prone to burnout. This longitudinal qualitative study explores the underlying drivers of burnout and its protective factors among early‐career physicians in Japan during this transitional phase.

**Methods:**

At an urban Japanese hospital, seven residents completed semi‐structured interviews at the end of their protected two‐year basic postgraduate clinical training (2020) and again after transitioning to specialty training (2022); one further resident provided only partial information. Twelve senior physicians responsible for resident education and support center staff were interviewed for triangulation. Data were analyzed using grounded theory techniques with NVivo 14.

**Results:**

No participant described experiences consistent with burnout during basic postgraduate clinical training, as residents perceived their environment as highly protected, often reporting a lack of primary responsibility and a passive “guest‐like” behavior rooted in the Japanese cultural desire to remain “good students.” However, follow‐up interviews revealed that the abrupt transition to independent practice exposed them to heavy responsibilities and complex patient management; burnout‐like episodes after the transition were described by some participants. Overcoming manageable challenges with adequate support was described as contributing to professional growth. Peer communication and continuous mentoring were vital mitigating factors.

**Conclusion:**

Burnout‐like experiences among early‐career physicians appear to be driven mainly by the abrupt shift in clinical responsibility during the transition to independent practice rather than the absolute workload during initial training. Medical education programs should consider structured “graduated autonomy” and continuous mentoring to support this vulnerable transition.

## 1 Introduction

The term “burnout” was first described by Freudenberger in 1974 [1] and later theoretically framed by Maslach, who conceptualized it as a syndrome characterized by emotional exhaustion, depersonalization, and reduced personal achievement [2]. Today, burnout is recognized globally as a critical, work‐related condition that disproportionately affects healthcare professionals [1, 2]. It is not merely an individual psychological burden but a systemic threat that leads to increased medical errors, decreased patient satisfaction, and potential disruptions to healthcare delivery systems. A comprehensive systematic review revealed that a significant majority of physicians experience burnout symptoms at some point in their careers [3].

Early‐career physicians and residents are particularly susceptible to profound stress as they navigate the demanding early stages of their professional development. Systematic reviews focusing on postgraduate medical trainees indicate high global prevalence rates of burnout [4]. Factors such as overwork, poor work environment, heavy job demands, and conflicts regarding work‐life balance have been consistently identified as significant risks for emotional exhaustion during residency training [4, 5]. However, training systems differ significantly across countries, suggesting that the drivers and manifestations of burnout may vary depending on the specific educational context and structural environment.

The postgraduate residency training system in Japan provides a unique context for this issue. It mandates a two‐year clinical training program where residents rotate through multiple specialties before selecting a definitive career path, exposing them to fast‐paced environmental changes and continuous relationship‐building with various medical staff. During this period, residents practice under the supervision of attending physicians and rarely hold primary responsibility for patient care. Completion of the two‐year program is a prerequisite for independent practice within the Japanese national health insurance system; in practice, physicians first assume primary and substantially independent responsibility for patient care upon entering specialty training in their chosen field. The “transition to independence” examined in this study therefore refers to the move from this supervised rotational training to specialty training. Previous quantitative studies in Japan have reported varying burnout prevalence rates among residents, ranging from 18% to 33% [6, 7, 8]. While these studies have identified factors such as long working hours and low autonomy [8], the majority of this existing research is highly heterogeneous, cross‐sectional, and lacks the capacity to extract causal relationships. Crucially, they overlook the longitudinal dynamics during the most vulnerable phase of a young physician's career: the abrupt transition from the highly protected, general rotational residency to assuming primary, independent responsibility in a specific clinical specialty.

To address this gap, this qualitative study aimed to investigate the underlying drivers of burnout and the protective factors that mitigate it among early‐career physicians in Japan through a longitudinal approach. By conducting in‐depth interviews with a cohort of residents at the end of their protected two‐year training and following up as they transitioned into specialty practice, we hypothesized that the shifting balance of clinical responsibility, autonomy, and environmental support critically influences their mental well‐being and professional maturation.

## 2 Methods

### 2.1 Research Setting

This qualitative study examined the occupational well‐being of residents at a city hospital in Japan (Hospital A). Throughout this manuscript, following the terminology of Nishigori et al. [9], we use “basic postgraduate clinical training” to refer to the mandatory two‐year rotational training program and “specialty training” to refer to the subsequent specialty training program. “Residents” refers to physicians undergoing basic postgraduate clinical training, and “senior physicians” refers to physicians who had completed specialty training and were responsible for resident education at the study hospital. Included participants were limited to second‐year postgraduate residents who had completed their clinical training at the urban hospital with more than 400 rooms for inpatients. There are 8 residents per year. At Hospital A, each resident is assigned a mentor through consultation between the resident support center and the resident; mentors are typically mid‐career physicians, often selected from the department of the resident's first rotation, and the mentoring relationship is maintained throughout the two‐year training period. The program includes formal one‐on‐one meetings approximately every three months, hospital‐funded informal dinner meetings, and day‐to‐day informal support. Mentoring covers both career counseling and mental health support, and screening for depressive symptoms is mandatory. There were no selection/exclusion criteria based on age, known past medical history such as mental illness, or monthly working hours. To ensure balance of the characteristics of the residency environment, residents who had experienced COVID‐19 during the same academic year were identified as the primary target group. Senior physicians were recruited through snowball sampling, whereby initial participants and the clinical training support center referred additional senior physicians involved in resident education, typically the residents' mentors and supervising physicians. We also investigated residents who had completed clinical training at the same hospital and senior physicians responsible for resident education in our hospital to speculate this theme from different perspectives. Recruitment continued until data saturation was achieved. Saturation was assessed by the PI concurrently with data collection and analysis and was judged to have been reached when successive interviews generated no new codes or themes; this judgment was reviewed and confirmed by the research team.

### 2.2 Data Collection

Semi‐structured in‐depth interviews were conducted in Japanese, face‐to‐face in a private room within the hospital, with only the interviewer and the participant present. Interviews were recorded but participants could decline the recording. Data were not collected when the possible participant declined. The interview consisted of 10 questions on six topics. It covered background and professional views, training systems, stress experiences, burnout concepts, mentoring, and COVID19. Because no formal burnout assessment instrument was administered, participants' accounts were treated as subjective descriptions of stress, exhaustion, and loss of motivation, and are distinguished throughout this manuscript from burnout as a formally defined construct. A semi‐structured interview guide was originally developed for this study by the authors (see File S1). The same interview guide was used at both the 2020 and 2022 interview waves. Initial interviews were conducted by the principal researcher in March 2020, at the end of the participants' basic postgraduate clinical training, and follow‐up interviews were conducted in March 2022, after the participants had transitioned to specialty training. Each interview was intended to last 30–45 min. Residents were invited face‐to‐face by the PI before the completion of their training, and consent was obtained during the training period; and data analysis, including transcript checking and coding, was performed within one month after each interview wave. Clinical residents who participated in the interviews were not compensated; however, refreshments were provided at the principal investigator's expense.

### 2.3 Data Analysis

Interviews were transcribed in Japanese by the principal investigator, partly manually and partly using a commercially available automatic speech‐recognition service, as NVivo did not support Japanese speech recognition at the time. All transcripts were checked against the audio recordings and corrected for recognition, language, and grammatical errors by the principal investigator. Based on the three‐dimensional conceptualization of burnout by Maslach [2] and prior studies of clinician burnout [3, 4, 5, 6, 7, 8, 10], a preliminary code set was developed, organized along the six topics of the interview guide. This framework constituted the pre‐created conceptual model, linking hypothesized drivers and mitigators of burnout (e.g., workload, responsibility, autonomy, mentoring, and peer support) to the interview topics. Coding of the completed transcripts was performed using NVivo14. Quotations presented in this manuscript were translated from Japanese into English by the PI. The analysis was informed by grounded theory techniques, employing multiple iterative cycles of coding and constant comparison across interviews and time points. We iteratively applied codes deductively derived from a pre‐created conceptual model and codes inductively derived from a detailed reading of the interview transcripts. All transcripts were coded by the principal investigator (M.W.). The full code set and the correspondence between codes and participant quotations were reviewed by a senior co‐author (Y.H.), and disagreements were resolved through discussion. The research team then reviewed key themes and corresponding quotes to assess the evidence supporting the themes, and we developed final concepts with insights from the clinical training support center in our hospital. Consensus was made through discussion. The analytic chain linking illustrative quotations, codes, categories, and themes is presented in File S2.

### 2.4 Reflexivity and Positionality

The principal investigator (PI) is a male physician specializing in rheumatology at the study hospital, who completed his own basic postgraduate clinical training at a different institution. The PI received formal training in qualitative methods—including study design, interpretation, and coding—as part of his Master of Public Health program at the Johns Hopkins Bloomberg School of Public Health. The PI has personal experience of burnout, which motivated this research and was explicitly acknowledged as a potential source of interpretive bias throughout the analysis. Eight of the 12 senior physician participants belonged to the PI's specialty and were his colleagues or superiors at the study hospital, and the PI had served as a direct clinical supervisor for some of the resident participants; thus, an “insider” relationship existed between the interviewer and many participants. This positionality facilitated a deep understanding of the specific clinical context, jargon, and implicit workplace culture, allowing for the establishment of strong rapport during interviews. To mitigate potential biases arising from shared professional backgrounds and existing hierarchical relationships, the interviewer consciously maintained a neutral stance, encouraging participants to elaborate on their experiences rather than assuming shared understanding. Participants were assured that participation was voluntary and confidential and would have no bearing on training evaluations or workplace relationships. Data analysis was iteratively reviewed to ensure that the themes were grounded directly in the participants' narratives rather than the researcher's preconceived notions.

## 3 Results

All eight eligible second‐year residents were invited to participate. Seven completed the full interview; one resident consented but declined recording and provided only partial information; these data were not coded or quoted, but were referred to as contextual field notes. Twelve senior physicians responsible for resident education also participated. Initial interviews were conducted in March 2020, at the end of the residents' basic postgraduate clinical training, and follow‐up interviews were conducted in March 2022, after the transition to specialty training. The seven residents included in the analysis consisted of three males and four females (age range, 26–29 years); all participants were of Asian ethnicity, most of the senior specialty was rheumatology, and all were in charge of resident education; other details are shown in Table 1. All seven residents who completed the initial interview also completed the follow‐up interview in March 2022. One resident reported experiencing a burnout episode between the first and second interviews.

**TABLE 1:** Demographic and professional characteristics of the participating residents (*N* = 7) and senior physicians (*N* = 12).

Category	Demographics	Resident doctors (*N* = 7)	Senior doctors (*N* = 12)
Age	26–29	7	2
	30–39	0	7
	40–	0	3
Gender	Male	3	7
	Female	4	5
Race and Ethnicity	Asian	7	12
Graduation	National University	4	10
	Private University	3	2
Specialty	General Internal Medicine	2	2
	Rheumatology	0	8
	Gastroenterology	2	2
	Pulmonology	1	0
	Obstetrics and gynecology	1	0
	Thoracic Surgery	1	0

Findings are reported in three parts: themes from the initial interviews at the end of basic postgraduate clinical training (T1, March 2020), themes from the follow‐up interviews during specialty training (T2, March 2022), and an integrated longitudinal interpretation. Across the two time points, four thematic areas were identified: reflections on training, the concept of burnout, professionalism and mentoring, and well‐being and protective factors.

Informative excerpt from the transcription was summarized in Table 2.

**TABLE 2:** Representative interview excerpts illustrating the four thematic areas identified in the analysis.

Category	Excerpts
1. Reflections on training	“Honestly, I can say that the residents have no authority in our hospital.” “It's like, whether you do something or not, you feel like you're in the way…” “Residents are somewhat treated as ‘guests’ here; we tend to lack initiative because we aren't given real authority.” “I think two years residency isn't necessarily a time when we can just do what we want to do…” “There was a gap between what I wanted to learn and what the senior doctors wanted me to learn…” “If we had more opportunities to interact repeatedly within the same ward, we could build better relationships… Having social gatherings or joint training sessions would make it easier to ask for help, which would significantly alleviate interpersonal stress.”
2. The Concept of Burnout	“I have absolutely no idea… but it never occurred to me…” “Is it like, after trying too hard, you end up unable to do anything?…” “It's probably more that we haven't burned brightly enough to actually burn out; there simply isn't enough fire [responsibility] during the residency.” “It would be good to notice by yourself, but it's hard to recognize…” “The initial trigger was a severe case that didn't go well while I was managing the patient as the primary attending… Being cornered by that heavy responsibility meant my own self‐study stalled, and I completely lost my mental bandwidth and sense of time. It became a vicious cycle.” “I actually feel that I've become stronger after experiencing it… Now, I even use my burnout as an icebreaker, telling junior residents, ‘I've burned out before, so feel free to talk to me about anything.’”
3. Professionalism and Mentoring	“Hard‐working residents get opportunities to skill‐up, or senior doctors are reluctant to guide unless they feel our enthusiasm…” “…like we won't get scolded, and seniors protect us, for better or worse…” “With fewer patients due to COVID19, we were told we're growing slowly… made me wonder if it's OK… We don't need to consider much about differential diagnoses…” “We may not busy because of outbreaks, but the hardship is still there.” “It was highly stressful dealing with the stigma and the judgmental looks from others, as if I had caused the outbreak, even though it wasn't at all clear that I was the source.”
4. Well‐being and protective factors	“Perhaps finding a balance by allowing for some imperfection is the key…” “Rather than confronting, it's like cleverly finding ways to slack off…” “I would say that working steadily is important… I also feel that taking enough break helped me continue working…” “It's about providing breaks systematically. Not just suddenly telling someone to take a few days off because they look exhausted, but ensuring that regular rest periods are built into the schedule.” “I feel that the senior doctors worked around the clock… many residents would find it hard to say they want a break while seeing their seniors working…”

### 3.1 Initial Interviews (T1, March 2020): A Protected Training Environment

At T1, residents felt that the workload in the emergency room (ER) had been hard, especially in the first year. The main sources of stress were the night shift, insufficient ER staffing, the fear of misdiagnosis, and responsibility for tasks. Their roles changed over time, and they were required to manage the night shift. Rotating through each specialty, they were aware that they could not gain learning opportunities without demonstrating motivation; however, they found it difficult to show initiative in decision‐making. Their conflicts were expressed in the word “guest.” (“Residents are somewhat treated as ‘guests’ here; we tend to lack initiative because we aren't given real authority.”) Although attending physicians tried to show consideration for residents' work, residents were not delegated authority and, as a result, simply followed the attending physicians. There was little awareness of exhaustion leading to burnout, and a common perception that they were protected from excessive work. Indeed, most residents were not familiar with the definition of burnout, describing it as “too much effort and exhaustion” without noting depersonalization or reduced personal accomplishment, and none reported experiences they identified as burnout during their training. Many residents expressed eagerness for personal growth and motivation toward future professionalism, yet showed a passive attitude of acquiring knowledge rather than making their own choices and decisions. Experiencing a patient's sudden deterioration or receiving negative feedback on one's medical decisions were typical stress events but were recognized as necessary for professional growth. Almost all residents had found a mentor and set milestones for themselves; each resident at Hospital A was assigned a mentor by the resident support center, and this system generally worked well. Residents regarded the most appropriate mentor as a doctor who was neither too close nor too far in age, with whom they could share empathy and consult easily. Regarding well‐being, residents indicated that its promotion included not only stress‐reduction methods such as sleep and rest but also awareness of one's general well‐being, self‐understanding, and the ability to communicate with peers and family; taking regular breaks and talking about trivial things with peers contributed to stress reduction. (“It's about providing breaks systematically. Not just suddenly telling someone to take a few days off because they look exhausted, but ensuring that regular rest periods are built into the schedule.”) The COVID‐19 pandemic, which reached the study hospital in the final months of their training, mainly reduced communication with other medical staff and disrupted smooth practice (“If we had more opportunities to interact repeatedly within the same ward, we could build better relationships… Having social gatherings or joint training sessions would make it easier to ask for help, which would significantly alleviate interpersonal stress.”), decreased opportunities to diagnose common febrile diseases and to participate in scheduled operations, and generated stigma toward residents affected by COVID‐19. (“It was highly stressful dealing with the stigma and the judgmental looks from others, as if I had caused the outbreak, even though it wasn't at all clear that I was the source.”) Some residents noted that restrictions on social activities inversely provided time for rest, whereas those who took the “COVID‐19‐associated restraint” at face value were likely to suffer a loss of well‐being.

### 3.2 Follow‐Up Interviews (T2, March 2022): The Transition to Independent Practice

At T2, participants described that their duties had changed drastically since basic postgraduate clinical training. They experienced heavy responsibilities and complex patient management, including explanations to patients and their families and on‐call duties, and some reported that the workload of senior medical staff was greater than that of residents. While each stressor was eventually overcome, many participants believed that they were unable to keep track of their peers' stress. All participants had assumed primary attending responsibility immediately upon entering specialty training, and some explicitly stated that a more gradual increase in the intensity of responsibility and stress would have been preferable. (“There was this unspoken assumption that you would be on your own from day one; [senior physicians] do help you, but the responsibility is heavy.”) Burnout‐like experiences after the transition—complete exhaustion or loss of drive and motivation—were described by only one or two participants; the following detailed account derives primarily from a single participant and should therefore be regarded as an exploratory, illustrative case rather than a general pattern. This participant reported an episode of burnout in the year following training and experienced a temporary leave from work. While caring for a critically ill patient, the patient's gradually worsening condition induced feelings of helplessness and a loss of confidence, and managing and interacting with the patient's family required considerable time. (“The initial trigger was a severe case that didn't go well while I was managing the patient as the primary attending… Being cornered by that heavy responsibility meant my own self‐study stalled, and I completely lost my mental bandwidth and sense of time. It became a vicious cycle.”) This physician reflected that excessive dedication to the profession and the heavy responsibility associated with decision‐making had been the primary causes of the episode, and was able to return to work after adequate rest, retrospectively perceiving that the experience had made them stronger. (“I actually feel that I've become stronger after experiencing it… Now, I even use my burnout as an icebreaker, telling junior residents, ‘I've burned out before, so feel free to talk to me about anything.’”) Participants continued to emphasize the importance of adequate rest, peer communication, and mentoring, while noting that physicians with low interactive skills and a poor grasp of their own capacities were unlikely to call for help.

### 3.3 Longitudinal Integration Across the Two Time Points

Comparing the two time points revealed a marked longitudinal contrast. At T1, residents described a protected, low‐responsibility environment in which they behaved as “guests” and rarely perceived exhaustion leading to burnout; their orientation was that of learners adjusting themselves to others, consistent with the Japanese tendency to “read the situation” and to regard harmony as a virtue. At T2, after transitioning to specialty training, the same participants described an abrupt increase in primary responsibility and decision‐making authority, and it was in this period—not during the protected training—that burnout‐like experiences were reported. Protective factors were consistent across both time points: peer communication, adequate and systematically scheduled rest, and accessible mentoring were repeatedly described as vital, whereas their disruption—by COVID‐19‐related communication restrictions at T1, or by isolation in new workplaces at T2—was described as a threat to well‐being. Taken together, the longitudinal data suggest that the driver of burnout‐like experiences in this cohort was the abrupt shift in clinical responsibility at the transition to independent practice rather than the absolute workload during training, and that continuity of peer and mentoring support across this transition was perceived as the key protective factor.

## 4 Discussion

This longitudinal qualitative study followed a single cohort of Japanese physicians across the transition from basic postgraduate clinical training to specialty training. Three qualitative findings stand out. First, at T1, residents described a highly protected training environment in which they held little primary responsibility and behaved as “guests” lacking clinical authority, consistently shielded by senior staff. Second, at T2, after the transition to specialty training, the same participants described an abrupt assumption of primary responsibility, and burnout‐like experiences were reported only in this period. Third, peer communication, systematically scheduled rest, and accessible mentoring were consistently described as protective at both time points. Notably, no participant described experiences that they identified as burnout during the training period itself; given the small, single‐institution sample and the absence of a validated burnout measure, this observation reflects participants' subjective accounts and should not be interpreted as a prevalence estimate. Previous quantitative studies in Japan have reported burnout prevalence of 18%–33% among residents [7]; our longitudinal design complements such cross‐sectional metrics by tracing how perceptions of responsibility and stress evolved within the same individuals over time. Participants themselves noted that the protected environment lacked the “fire” of ultimate responsibility, which is typically required to test and build professional capacities.

To understand this contrast between the protected training environment and the post‐transition period, it is helpful to consider the sociocultural context of Japanese postgraduate training, which aligns with emerging literature highlighting the impact of regional cultural climates on Asian medical trainees [8]. The concepts introduced below—being “the one to be evaluated,” the “good student,” and the “cliff of independence”—are the authors' interpretive framings, developed to make sense of participants' narratives such as the “guest” metaphor, rather than terms used by the participants themselves. During the mandatory two‐year rotation, residents carry the constant burden of being “the one to be evaluated.” As a social factor, they are strongly expected to maintain harmonious relationships with peers and multiple professions, often prioritizing being a “good student” over taking proactive clinical risks. This cultural pressure further reinforces their passive, “guest‐like” behavior. Our longitudinal follow‐up revealed a stark contrast once these physicians completed this general rotation and transitioned to independent practice. The abrupt shift from a highly protected, rotational environment to assuming primary, solitary responsibility for complex patient care acted as a severe stressor. This sudden exposure—what we term the “cliff of independence”—was followed by burnout‐like experiences in some participants, suggesting that a heavily shielded residency leaves young physicians underprepared for the harsh realities of full autonomy. By contextualizing these experiences within the broader literature, our findings regarding the Japanese “good student” dynamic and subsequent transition shocks provide a valuable, culturally specific illustrative example that expands the international discourse on early‐career physician well‐being. Our findings also resonate with the emerging literature on patient care ownership. The “guest” experience described by our participants appears to encompass several distinguishable components: limited autonomy in decision‐making, limited ownership of patient care, limited entrustment by supervisors, and a hierarchical workplace culture. In a multicenter study across Japan, patient care ownership among trainees increased with seniority and with the perceived quality of the learning environment [11], and qualitative work in pediatric residency suggests that supervisors' behaviors modulate residents' sense of ownership and of “having a voice” in patient care [12]. Notably, the Japanese validation of the Patient Care Ownership Scale identified a distinct “sense of ownership” factor not observed in the original version, which its authors attributed to culturally specific codes of personal conduct [13]; this observation is consistent with our interpretation that culturally shaped role expectations reinforce the “guest” position of Japanese residents. From the perspective of entrustment decision‐making [14], the abrupt transition observed in our cohort may reflect the absence of deliberate, graduated entrustment: participants moved from a state of minimal entrusted responsibility during basic postgraduate clinical training directly to full clinical responsibility upon entering specialty training. This framing anchors our “graduated autonomy” recommendation in established competency‐based frameworks, in which supervision is progressively withdrawn along defined levels as trust is established.

Importantly, our findings should not be interpreted as suggesting that burnout itself fosters resilience. Burnout is an occupational phenomenon arising from workplace conditions, and physicians who experience it should be regarded as affected by those conditions. What participants described as promoting professional growth was the experience of overcoming manageable clinical challenges with adequate support—not the experience of burnout itself. Although one participant retrospectively perceived themselves as having become stronger after recovering from a burnout episode, this individual perception does not imply that burnout experiences are desirable or protective. This underscores the vital role of “eustress”—where an appropriate clinical load combined with genuine decision‐making authority is essential for professional maturation. While previous reports have explored the association between long working hours and burnout [10], our findings generate the hypothesis that limiting working hours alone, without concurrently providing graduated autonomy, may not be sufficient to support long‐term professional development. Professional growth appeared to be supported not by the complete absence of stress, but by successfully overcoming manageable clinical challenges with appropriate support.

These findings have direct and practical implications for postgraduate medical education. Rather than merely protecting residents from workload, training programs should consider implementing structured “graduated autonomy” to help bridge the gap between initial residency and independent practice. Furthermore, longitudinal mentoring and peer support systems must be intentionally extended beyond the early training period to target this highly vulnerable transition phase. This study has several limitations. First, as a single‐center qualitative study conducted at an urban hospital with a small sample, the findings are subject to selection bias, and their transferability to other healthcare settings is limited. Second, recall bias and social desirability bias may have influenced participants' accounts, particularly given the two‐year interval between interviews and the hierarchical and collegial relationships between the interviewer and participants described in the Reflexivity section. Third, the predominance of rheumatologists among the senior physician participants may have skewed the educators' perspectives toward a single specialty, and one resident participated only partially. Fourth, burnout‐like experiences after the transition were described by only one or two participants, so the concept of delayed burnout generated by this study should be regarded as exploratory. Fifth, transcripts and findings were not returned to participants for confirmation (member checking), and the English translations of quotations were not independently verified. Finally, this study did not formally incorporate a quantitative measure, such as the Maslach Burnout Inventory (MBI). Although structured psychological scores offer standardized metrics for symptom levels, the primary objective of this research was to longitudinally capture the dynamic, nuanced subjective experiences and shifting perceptions of clinical responsibility that are often obscured by cross‐sectional quantitative data. Furthermore, given that the psychometric validation of the Japanese version of such standardized scales among medical residents remains a subject of ongoing methodological discussion in the domestic literature, we prioritized a rigorous longitudinal qualitative design. This approach successfully illustrated the vulnerable transition phase from a shielded general rotation to independent specialty practice, providing deep contextual insights that quantitative metrics alone cannot fully elucidate.

## 5 Conclusion

This longitudinal qualitative study traced Japanese physicians from the end of their protected two‐year basic postgraduate clinical training into specialty training. During training, participants described a protected, “guest”‐like position with little primary responsibility, and none described experiences they identified as burnout; after the transition, they described an abrupt assumption of primary responsibility, and burnout‐like experiences emerged in some participants. Peer communication, systematically scheduled rest, and accessible mentoring were consistently described as protective across both periods. These findings suggest that the abrupt shift in clinical responsibility at the transition to independent practice—rather than the absolute workload during training—poses the greatest threat to early‐career physicians' occupational well‐being, and that because individual capacities and support needs vary, uniform administrative interventions alone, such as strictly capping working hours, may not be sufficient.

To help prevent burnout‐like experiences during this critical transition, our findings suggest that healthcare institutions may benefit from more individualized support systems. First, training programs should design structured “graduated autonomy” to optimize clinical workload, shifting the focus from merely limiting hours to managing the quality and responsibility of the work. Second, continuous, longitudinal mentoring should be established, extending intentionally beyond the initial training period to support physicians during their most vulnerable transition. Finally, institutions should implement regular subjective and objective evaluations of both the physician's well‐being and clinical load to detect early signs of emotional exhaustion. Educational stakeholders may need to shift their strategy from simply shielding young physicians toward equipping them with the resources and manageable challenges that may support long‐term professional growth.

## Author Contributions

**Mizuki Kato:** investigation, validation, writing – review and editing. **Yoichiro Haji:** investigation, validation, writing – review and editing, supervision, conceptualization. **Yukina Yokoyama:** investigation, validation, writing – review and editing. **Mitsuru Watanabe:** conceptualization, methodology, formal analysis, investigation, data curation, writing – original draft, writing – review and editing, project administration. **Kosuke Nakano:** writing – review and editing, validation, investigation. **Yui Amari:** investigation, validation, writing – review and editing.

## Funding

The authors have nothing to report.

## Ethics Statement

The study was conducted in accordance with the Declaration of Helsinki. The study protocol was approved by the ethical review board of Daido Hospital (ECD2022‐017, March 2022).

## Consent

Written informed consent was obtained from all individual participants included in the study.

## Conflicts of Interest

The authors declare no conflicts of interest.

## Supporting information


**File S1:** Questionnaire file. Semi‐structured interview guide (English version) developed for evaluating the psychological transitions, responsibilities, and burnout risks among early‐career physicians.

**File S2:** Coding framework. Analytic chain from illustrative quotations to codes, categories, and themes across the two interview time points (T1, March 2020; T2, March 2022).

## Data Availability

The data that support the findings of this study are available on request from the corresponding author. The data are not publicly available due to privacy or ethical restrictions.

## References

[1] H. Freudenberger and H. J. Freudenberger , “Staff Burnout,” Journal of Social Issues 30 (1974): 159–165.

[2] C. Maslach and S. Jackson , “The Measurement of Experienced Burnout,” Journal of Organizational Behavior 2 (1981): 99–113.

[3] L. S. Rotenstein , M. Torre , M. A. Ramos , et al., “Prevalence of Burnout Among Physicians: A Systematic Review,” Journal of the American Medical Association 320, no. 11 (2018): 1131–1150, 10.1001/jama.2018.12777. 30326495 PMC6233645

[4] L. Naji , B. Singh , A. Shah , et al., “Global Prevalence of Burnout Among Postgraduate Medical Trainees: A Systematic Review and Meta‐Regression,” CMAJ Open 9, no. 1 (2021): E189–E200, 10.9778/cmajo.20200068. PMC8034324 33688027

[5] A. Y. Zhou , M. Panagioti , A. Esmail , R. Agius , M. van Tongeren , and P. Bower , “Factors Associated With Burnout and Stress in Trainee Physicians: A Systematic Review and Meta‐Analysis,” JAMA Network Open 3, no. 8 (2020): e2013761, 10.1001/jamanetworkopen.2020.13761. 32809031 PMC7435345

[6] R. Miyoshi , H. Matsuo , R. Takeda , H. Komatsu , H. Abe , and Y. Ishida , “Burnout in Japanese Residents and Its Associations With Temperament and Character,” Asian Journal of Psychiatry 24 (2016): 5–9, 10.1016/j.ajp.2016.08.009. 27931906

[7] Y. Nishimura , T. Miyoshi , M. Obika , H. Ogawa , H. Kataoka , and F. Otsuka , “Factors Related to Burnout in Resident Physicians in Japan,” International Journal of Medical Education 10 (2019): 129–135, 10.5116/ijme.5caf.53ad. 31272084 PMC6766397

[8] P. Puranitee , F. F. C. J. Stevens , S. Pakakasama , et al., “Exploring Burnout and the Association With the Educational Climate in Pediatric Residents in Thailand,” BMC Medical Education 19, no. 1 (2019): 245. 31277615 10.1186/s12909-019-1687-7 PMC6612205

[9] H. Nishigori , “Reflections on the Reform of Postgraduate Medical Education in Japan,” Medical Teacher 46, no. S1 (2024): S4–S10, 10.1080/0142159X.2024.2372108. 39545499

[10] T. Matsuo , O. Takahashi , K. Kitaoka , H. Arioka , and D. Kobayashi , “Resident Burnout and Work Environment,” Internal Medicine 60, no. 9 (2021): 1369–1376, 10.2169/internalmedicine.5872-20. 33281158 PMC8170257

[11] H. Fujikawa , D. Son , T. Aoki , and M. Eto , “Association Between Patient Care Ownership and Personal or Environmental Factors Among Medical Trainees: A Multicenter Cross‐Sectional Study,” BMC Medical Education 22 (2022): 666, 10.1186/s12909-022-03730-y. 36076223 PMC9461127

[12] M. A. Robinson , J. L. Bowen , M. Aylor , and S. van Schaik , “Having a Voice: Resident Perceptions of Supervision, Decision‐Making and Patient Care Ownership,” Academic Pediatrics 24, no. 3 (2024): 519–526, 10.1016/j.acap.2023.10.012. 37951350

[13] H. Fujikawa , D. Son , K. Kondo , M. Djulbegovic , Y. Takemura , and M. Eto , “Translating and Validating a Japanese Version of the Patient Care Ownership Scale: A Multicenter Cross‐Sectional Study,” BMC Medical Education 21 (2021): 415, 10.1186/s12909-021-02853-y. 34344354 PMC8329902

[14] O. Ten Cate , D. Hart , F. Ankel , et al., “Entrustment Decision Making in Clinical Training,” Academic Medicine 91, no. 2 (2016): 191–198, 10.1097/ACM.0000000000001044. 26630606

