# Supplementary material for: The Transition to Independence: A Longitudinal Qualitative Study on Drivers of Burnout in Japanese Early‐Career Physicians
Source: J Gen Fam Med. 2026 Aug 2;27(5):e70159. doi: 10.1002/jgf2.70159 (PMC13430068; doi:10.1002/jgf2.70159)
Supplement: Supplementary file 1 — File S1: Questionnaire file. Semi‐structured interview guide (English version) developed for evaluating the psychological transitions, responsibilities, and burnout risks among early‐career physicians. [file JGF2-27-e70159-s001.docx]

**Supplementary File 1**

**SOCIO-DEMOGRAPHICS**

1. Assign ID:

2. Gender: Male/female

3. Age:

4.Education: Highest level of school completed

5.Household members

6.Club activity at university

7.Past Medical History/Medication

8.Alcohol/Smoking

9.Sleeping habit：Weekdays/Holidays

**INTERVIEW GUIDE**

INTRODUCTION
Thank you for talking with me today. We appreciate your time in helping us understand the experiences of Japanese residency and burnout. The information you provide today will remain completely confidential and will be helpful to design future programs to support future resident.

I’d like to hear your story in relation to your residency and burnout....start wherever you feel comfortable.

Background/Career view

1. 1Please tell us about your motivation/reason for wanting to become a doctor.

2. Do you have a specific department as your career for the future?

If there have been any changes during your residency, please tell us how things have changed.

Residency

3.Clinical residency is said to be very stressful; how do you feel about the burden of the current system?

4. Do you think the evaluation you are getting from the people around you is appropriate? Did you experience any conflicts with colleagues around you?

Own Experience

5. Did you experience any strong stress during your clinical residency?

6. How and how long did you recover from the stress?

7.Have you ever heard of the term "Burn Out"?

Have you ever experienced "burn out" or similar emotion?

Mentoring

8.Did you have someone you could talk to when you had problems? Have you found a role model during residnecy?

COVID19 pandemic

9. Did the COVID19 pandemic situation affect your life (diet, sleep, return home) and residency?

Others

Please tell me if you have any ideas to prevent Burn Out as an employer or senior doctor?
