# Supplementary material for: The Transition to Independence: A Longitudinal Qualitative Study on Drivers of Burnout in Japanese Early‐Career Physicians
Source: J Gen Fam Med. 2026 Aug 2;27(5):e70159. doi: 10.1002/jgf2.70159 (PMC13430068; doi:10.1002/jgf2.70159)
Supplement: Supplementary file 2 — File S2: Coding framework. Analytic chain from illustrative quotations to codes, categories, and themes across the two interview time points (T1, March 2020; T2, March 2022). [file JGF2-27-e70159-s002.docx]

# Supplementary File 2. Coding framework: analytic chain from illustrative quotations to codes, categories, and themes

*Codes and categories were verified by the authors against the original Japanese transcripts. Quotations were translated from Japanese into English by the first author.*

| Time point | Illustrative quotation (English translation) | Code | Category | Theme |
| --- | --- | --- | --- | --- |
| T1 | “Residents are somewhat treated as ‘guests’ here; we tend to lack initiative because we aren’t given real authority.” | Lack of delegated authority; passive followership | Limited autonomy and ownership during training (“guest” position) | Reflections on training |
| T1 | “In the emergency department, I had to manage patients who could deteriorate at any moment all on my own… constantly worrying, ‘What if I miss something?’—that was really hard at times.” | ER burden; solitary coverage; fear of misdiagnosis | Workload-related stress during training | Reflections on training |
| T1 | Burnout described as “too much effort and exhaustion” (without depersonalization or reduced accomplishment) | Partial understanding of burnout construct | Limited familiarity with burnout | The concept of burnout |
| T1 | “For residents who lack motivation in the first place, they probably feel that nobody teaches them anything, that they learn nothing, that it’s no fun—nothing but chores.” | Passive learning attitude; motivation-dependent learning opportunities | “Good student” orientation | Professionalism and mentoring |
| T1 | Mentor described as ideally “neither too close nor too far in age,” someone easy to consult (paraphrased) | Preferred mentor proximity; consultability | Mentoring structure and fit | Professionalism and mentoring |
| T1 | “If we had more opportunities to interact repeatedly within the same ward, we could build better relationships… Having social gatherings or joint training sessions would make it easier to ask for help, which would significantly alleviate interpersonal stress.” | Reduced informal communication (COVID-19); help-seeking barriers | Disruption of protective factors by COVID-19 | Well-being and protective factors |
| T1 | “It was highly stressful dealing with the stigma and the judgmental looks from others, as if I had caused the outbreak, even though it wasn’t at all clear that I was the source.” | COVID-19 infection stigma | Pandemic-specific stressors | Reflections on training |
| T1 | “It’s about providing breaks systematically. Not just suddenly telling someone to take a few days off because they look exhausted, but ensuring that regular rest periods are built into the schedule.” | Systematic, scheduled rest | Rest and recovery as protective factors | Well-being and protective factors |
| T1 | “Perhaps finding a balance by allowing for some imperfection is the key…” | Tolerance of imperfection | Self-understanding and coping | Well-being and protective factors |
| T2 | “The initial trigger was a severe case that didn’t go well while I was managing the patient as the primary attending… Being cornered by that heavy responsibility meant my own self-study stalled, and I completely lost my mental bandwidth and sense of time. It became a vicious cycle.” | Responsibility overload; vicious cycle of exhaustion | Burnout-like episode after transition | The concept of burnout |
| T2 | “I actually feel that I’ve become stronger after experiencing it… Now, I even use my burnout as an icebreaker, telling junior residents, ‘I’ve burned out before, so feel free to talk to me about anything.’” | Retrospective perception of strengthening; peer disclosure | Recovery and reintegration | Well-being and protective factors |
| T2 | “There was this unspoken assumption that you would be on your own from day one; (senior physicians) do help you, but the responsibility is heavy.” | Abrupt assumption of primary responsibility; preference for graduated intensity | Transition shock and graduated autonomy | Reflections on training |
| T2 | “When dealing with juniors, I do try to recall my own experiences and treat them accordingly.” | Supporting juniors by drawing on one’s own experience | Continuity of peer and near-peer support | Well-being and protective factors |

*Note: categories were derived by grouping codes; themes correspond to the four thematic areas reported in the Results. The deductive starting codes were drawn from the preliminary conceptual model (workload, responsibility, autonomy, mentoring, peer support); inductive codes (e.g., “guest,” stigma) emerged from the transcripts.*
